# Interactions Between BMP2/BMP4 Gene Polymorphisms and Fluoride Exposure on Essential Hypertension: A Cross-Sectional Study in China

**DOI:** 10.3390/toxics13020126

**Published:** 2025-02-08

**Authors:** Yue Gao, Qingbo Wang, Junhua Wu, Yang Liu, Xin Wang, Yanhui Gao, Yanmei Yang

**Affiliations:** 1 Center for Endemic Disease Control, Chinese Center for Disease Control and Prevention, Harbin Medical University, No. 157 Baojian Road, Nangang District, Harbin 150081, China; gao15663673888@163.com (Y.G.); wangqingbo0628@163.com (Q.W.); m13245380261@163.com (X.W.); 2 Key Lab of Etiology and Epidemiology, Education Bureau of Heilongjiang Province, Ministry of Health of P. R. China, Harbin Medical University, Harbin 150081, China

**Keywords:** fluoride, hypertension, BMP2/4, single nucleotide polymorphism, gene-environment interaction

## Abstract

(1) Objective: To evaluate the relationship between fluoride exposure, interactions of BMP2/BMP4 gene polymorphisms, and fluoride exposure on essential hypertension. (2) Methods: A cross-sectional study was conducted among 725 participants in a high-fluoride region of Shanxi Province, China. Urinary fluoride concentrations were measured as indicators of fluoride exposure. Hypertension was diagnosed based on standard guidelines. BMP2 (rs1005464) and BMP4 (rs17563) polymorphisms were genotyped. Logistic regression and interaction models were performed to evaluate associations and interactions between fluoride exposure, gene polymorphisms, and hypertension. (3) Results: Higher urinary fluoride concentrations were significantly associated with an increased risk of hypertension, exhibiting a dose-dependent relationship. The rs1005464 (G > A) polymorphism of BMP2 was identified as a protective factor against hypertension in individuals with the AG + AA genotype. Significant interactions were observed between the BMP2 rs1005464 and BMP4 rs17563 polymorphisms, influencing hypertension risk. Additionally, both multiplicative and additive interactions between high fluoride exposure and the BMP4 rs17563 polymorphism were identified, highlighting the combined impact of environmental and genetic factors on hypertension. (4) Conclusions: Fluoride exposure is positively associated with hypertension. BMP2 gene polymorphisms affect the risk of hypertension, and BMP4 gene polymorphisms may modify the impact of fluoride on hypertension.

## 1. Introduction

Fluorine, a naturally abundant element [1], exerts a multifaceted influence on human health, functioning as a double-edged sword. Within recommended limits, fluorine contributes positively to bone mineralization and enamel development [2]. Conversely, excessive intake may result in fluorosis [3], a condition that not only compromises bone tissues but also poses risks to non-osseous tissues, including the cardiovascular system [4]. Epidemiological studies have indicated a potential link between fluorine exposure and cardiovascular dysfunction, with evidence suggesting a correlation between fluorine levels and blood pressure [5]. Nevertheless, the nature of this association—whether positive or negative—remains contested and warrants further investigation.

Blood pressure is influenced by cardiac output and peripheral vascular resistance, with vascular resistance primarily determined by vessel diameter and blood viscosity. Vascular remodeling, characterized by vessel narrowing, wall stiffening, and loss of elasticity, is a key factor in the onset and progression of hypertension [6]. Vascular calcification, involving the deposition of calcium salts in the vessel walls, is a crucial mechanism in vascular remodeling, leading to hardening and elasticity loss. Additionally, vascular calcification and bone metabolism disorders may share common risk factors and underlying mechanisms [7]. Studies have shown that high fluoride exposure can disrupt bone metabolism [8], as fluoride enhances growth factors in bone cells and promotes osteoblast production via activation of the Wnt signaling pathway [9]. Bone morphogenetic proteins (BMPs), essential for osteoblast survival and differentiation, are implicated in this process. Research suggests that fluoride disrupts BMP expression, contributing to bone metabolism abnormalities [10], and BMP family members are recognized as key regulators in vascular calcification, capable of inducing the differentiation of vascular smooth muscle cells into osteogenic or chondrogenic-like cells [11]. Thus, BMPs could be important in cardiovascular disease or hypertension related to fluoride exposure.

Hypertension arises from the interaction between environmental and genetic factors. Among the most common heritable variations in humans are single nucleotide polymorphisms (SNPs), which represent DNA sequence polymorphisms caused by single nucleotide changes [12]. Over 30 genes and 1400 SNPs have been frequently linked to blood pressure regulation [13]. The BMP2/4 genes have been identified as potential candidates for genetic polymorphisms associated with hypertension. This study hypothesizes that specific polymorphisms in BMP2/4 may contribute to the susceptibility to hypertension, potentially modifying vascular function and response to environmental exposures such as fluoride [14]. While many studies have suggested a relationship between fluoride exposure and hypertension risk, few have provided epidemiological evidence on the specific role of BMP2/4 gene polymorphisms in this association. To address this gap, we conducted a study in Luliang City, Shanxi Province, an area with high fluoride exposure, aiming to determine (1) the relationship between fluoride exposure and hypertension; and (2) whether fluoride exposure and BMP2/4 genes polymorphism have interaction effects on hypertension. Given the potential role of BMP signaling in vascular health and the increasing concern regarding environmental fluoride exposure, this research is timely and important. By identifying genetic markers that may predispose individuals to hypertension in the context of fluoride exposure, this study could inform future preventive strategies and therapeutic interventions targeting genetic risk factors for hypertension.

## 2. Materials and Methods

### 2.1. Data Source

In 2018, a cross-sectional study was conducted in three villages in Lüliang City, Shanxi Province, where records of drinking water fluoride levels were available. A cluster sampling method was employed to select the villages. Trained staff administered standardized questionnaires and conducted clinical examinations for all participants. The standardized questionnaire collected information on demographic characteristics (age and sex) and lifestyle habits (such as alcohol consumption and smoking). Clinical examinations included measurements of blood pressure, height, and weight. Participants had to be (1) Han ethnicity, (2) aged 16 years or older, (3) residing continuously in the area for at least 10 years, and (4) providing informed consent to participate. Exclusion criteria were as follows: (1) individuals with missing samples or unmeasured indicators; (2) incomplete epidemiological data; (3) undetectable indicators; (4) other types of hypertension except essential hypertension; and (5) pregnant individuals. Participants aged ≥60 years were classified as elderly. Individuals who smoked at least one cigarette per day (currently or in the past) were categorized as smokers, while those who consumed alcohol more than twice a week (currently or in the past) were categorized as drinkers. Body mass index (BMI) was calculated as weight (kg) divided by the square of height (m^2^). Based on the BMI classification for Asian populations [15], participants were categorized as normal weight (BMI < 23.9) or overweight (BMI ≥ 24). All participants provided written informed consent.

### 2.2. Hypertension Diagnosis

Each participant’s blood pressure was measured twice in a seated position using a mercury sphygmomanometer after resting for at least five minutes, with the average of the two measurements recorded. Hypertension was diagnosed according to the 2020 International Society of Hypertension Global Hypertension Practice Guidelines, defined as a systolic blood pressure (SBP) ≥ 140 mmHg and/or diastolic blood pressure (DBP) ≥ 90 mmHg [16] or a history of hypertension medication use.

### 2.3. Urinary Fluoride (UF) Concentration Determination

Urine samples were stored at −20 °C before analysis. The fluoride concentration in urine was quantified using a fluoride ion-selective electrode (Yingke Crystal Materials Co., Changsha, China), measuring two independent aliquots for each sample, with the average of the two measurements representing the final fluoride concentration. Urine samples were processed and analyzed following standardized methods (WS/T 89-2015, China [17]).

### 2.4. Detection of Gene Locus Polymorphisms

A 5 mL blood sample was collected from the fasting arm vein and placed in an anticoagulant tube. After one hour at room temperature, the plasma was centrifuged at 3000 rpm for 10 min and then stored at −80 °C for genomic DNA analysis. DNA samples with concentrations greater than 20 μg/mL were genotyped using multiplex PCR and sequencing techniques by Beijing Xinjiyuan Biotechnology Co., Ltd. (Beijing, China). The primer sequences were as follows:

rs1005464: forward-5′-GTTTCTGTGAACGTCTAACTTACC-3′
reverse-5′-AATCAGTTAGTGGCCTTAAGAAAG-3′
extended-5′-CTTTCTTAAGGCCACTAACTGATT-3′
rs17563: forward-5′- ATAAATGTTTATACGGTGGAAGCC-3′
reverse-5′-ATATGCTTTTTCTTTTCCCCTTCC-3′
extended-5′-GGAAGGGGAAAAGAAAAAGCATAT-3′

### 2.5. Statistical Methods

This work included statistical analysis and visualizations utilizing SPSS 26.0 (IBM, Armonk, NY, USA) and RStudio (version 2023.03.0). The Kolmogorov–Smirnov test was employed to evaluate the normality of continuous variables. For non-normally distributed data, the Mann–Whitney U test was employed to detect differences between groups. Differences across groups for categorical data were assessed utilizing the chi-squared test. A binary logistic regression model was used to assess hypertension risk in the second, third, and fourth quartiles compared to the first quartile. The Hardy–Weinberg equilibrium test was conducted for SNP loci. When the genotype frequency distribution in the control group adhered to the Hardy–Weinberg equilibrium, it suggested that the sample population in this study was randomly selected and representative of the natural population. Genetic models were analyzed using SNPStats, an online software (https://www.snpstats.net/start.htm, accessed on 10 December 2024). The Akaike information criterion (AIC) and Bayesian information criterion (BIC) are two widely used criteria for selecting the optimal model, with lower AIC and BIC values indicating the best genetic models for our population’s SNP data. Binary logistic regression was further used to calculate hypertension risk across different populations and to analyze the combined effect of SNPs and varying urinary fluoride levels on hypertension risk. An additional binary logistic regression model was applied to assess the impact of two-SNP combinations on hypertension risk, incorporating the product term of the two loci to evaluate multiplicative interaction. Additive interaction between the two loci was evaluated by the relative excess risk due to interaction (RERI) and the synergy index (SI). A *p*-value < 0.05 was considered statistically significant.

## 3. Results

### 3.1. Characteristics of the Participants

The research comprised 725 people, 238 of whom were male and 487 of whom were female. Among them, 368 individuals were diagnosed with hypertension. The distribution of gender between the hypertension and control groups was not significantly different. The hypertension group had significantly higher age, BMI, and urinary fluoride concentrations compared to the control group. No substantial variations in smoking and drinking status were seen between the hypertension and control groups. Additionally, higher age, BMI, and urinary fluoride concentrations were correlated with a higher likelihood of hypertension (see Table 1). In addition, a comparison of characteristics was performed between the included and excluded participants from the 989 eligible subjects. No statistically significant differences were found in characteristics including age, gender, BMI, urinary fluoride concentration, smoking, and alcohol consumption between the two groups (see Table S1).

### 3.2. The Relationship Between Urinary Fluoride Concentration and the Risk of Hypertension

The association between urinary fluoride levels and the risk of hypertension was assessed using logistic regression analysis. Specifically, for each 1 mg/L increase in urinary fluoride concentration, the risk of developing hypertension was found to escalate by a factor of 0.307. Furthermore, after adjusting for potential confounding factors, when urinary fluoride levels were categorized into quartiles within the model, the third and fourth quartiles exhibited a significantly increased risk of hypertension compared to the first quartile (defined as <0.87 mg/L) (OR = 1.513 and OR = 1.647, respectively). Moreover, in the trend test, both before and after adjusting for confounding factors, the risk of hypertension increased with higher urinary fluoride concentrations (see Table 2).

### 3.3. Associations of Risk of Hypertension and BMP2/BMP4 Genotype

The genotype frequency distribution in the control group adhered to the Hardy–Weinberg equilibrium. We employed the SNPStats online software to examine the correlation between various genotypes and hypertension throughout five genetic models: codominant, dominant, recessive, overdominant, and log-additive. Analysis identified a significant association at the rs1005464 of the BMP2 and hypertension risk, with AIC and BIC criteria confirming a dominant inheritance pattern. The AG + AA genotype identified as a protective factor against hypertension. In contrast, the different genotypes of the rs17563 locus of the BMP4 gene did not show statistically significant associations with hypertension (see Table 3).

### 3.4. Stratified Analysis by Potential Risk Factor of Hypertension

A statistically significant disparity in hypertension prevalence was discovered between the GG and the AG + AA genotype group at the rs1005464 (χ^2^ = 4.115). This difference was also evident among individuals younger than 60 years and among smokers (χ^2^ = 4.152 and χ^2^ = 9.179, respectively), as shown in Table 4. A stratification analysis examined the association of rs1005464 with the risk of hypertension across subgroups defined by potential risk factors, including age, gender, BMI, urinary fluoride, cigarette smoking, and alcohol drinking, under the assumption of a dominant genetic model. The AG + AA genotype reduced the risk of prevalence of hypertension among individuals younger than 60 years (OR = 0.582), women (OR = 0.667), and alcohol drinkers (OR = 0.296). These results are shown in Table 5.

### 3.5. Gene–Gene Interactions on the Risk of Hypertension

The crossover analysis and interaction results between rs1005464 of BMP2 and rs17563 of BMP4 are indicated in Table 6. Regardless of whether confounding factors were adjusted, multiplicative and additive (RERI = 0.77 and RERI = 1.13, respectively) interactions were observed between the two SNPs. After adjusting for confounding factors, the rs1005464 (GG) and rs17563 (AG + GG) combination was associated with an increased risk of hypertension compared to the reference combination of rs1005464 (AG + AA) and rs17563 (AA) (OR = 1.99) (see Table 6).

### 3.6. Interactions Between Urinary Fluoride and Single Gene Polymorphism on the Hypertension Risk

High fluoride exposure and genotypes at rs17563 of the BMP4 gene exhibited both multiplicative and additive interactions (pre-adjustment RERI = −1.35; post-adjustment RERI = −1.38). After adjusting for confounders, the rs17563 (AG + GG) genotype combined with high fluoride exposure increased the risk of hypertension 1.21-fold compared to the reference group (rs17563 [AA] with high fluoride exposure, OR = 2.21) (see Table 7). In contrast, no significant multiplicative or additive interactions were observed between UF and rs1005464 of BMP2 (Table 8). However, after adjusting for confounders, high fluoride exposure combined with the rs1005464 (GG) genotype was associated with a 0.95-fold increased hypertension risk compared to the reference group (rs1005464 [AG + AA] with low fluoride, OR = 1.95).

## 4. Discussion

Fluorine, the thirteenth most abundant element on Earth, is highly accessible to the human body due to its chemical properties and geographic mobility [18]. Prior research suggests a potential link between fluoride exposure and an increased likelihood of vascular dysfunction and hypertension [19]. Previous studies investigating the correlation between fluoride exposure and blood pressure have faced several limitations, including small sample sizes, inadequate case numbers, diverse types of fluoride exposure, numerous confounding factors, and inconsistent inclusion criteria for hypertension cases. These issues have resulted in conflicting findings, with reports of positive, negative, and null associations. In our study, we addressed these limitations by investigating residents of Wenshui County, Lüliang City, Shanxi Province, who had lived in the area for at least 10 years, were of Han ethnicity, were aged 16 years or older, and had no other forms of hypertension. Potential confounding factors were rigorously controlled. Moreover, there were no statistically significant differences in the general characteristics between the included and excluded populations in this study, indicating that the study population is representative and can accurately reflect the target population. For this study, we selected urinary fluoride levels as an indicator of fluoride exposure. Regardless of whether urinary fluoride is analyzed as a continuous variable, a categorical variable, or analyzed for trend, the findings consistently demonstrate a statistically significant positive association between urinary fluoride concentration and hypertension prevalence. Notably, after adjusting for confounding factors, each 1 mg/L increase in urinary fluoride was associated with a 21% increase in hypertension risk. Additionally, urinary fluoride concentrations above 1.25 mg/L were linked to an elevated risk of hypertension, suggesting a dose-dependent effect with a potential threshold beyond which fluoride exposure may significantly raise blood pressure. This finding aligns with multiple animal studies supporting fluoride’s impact on hypertension [20,21].

Bone morphogenetic proteins (BMPs), part of the TGF-β family, play key roles in regulating cell proliferation, differentiation [22], and apoptosis [23]. Dysregulated BMP signaling contributes to skeletal and cardiovascular diseases, including hypertension [24]. BMP2 and BMP4 are particularly important in vascular smooth muscle cell (VSMC) trans-differentiation, a process that leads to the loss of muscle characteristics, increased expression of bone-related proteins, and vascular calcification, all of which are linked to hypertension [25]. Polymorphisms in BMP2 and BMP4 genes, such as rs235756 (BMP2) and rs17563 (BMP4), have been associated with cardiovascular and skeletal diseases. A study on middle-aged Finnish men found a significant association between the BMP2 gene variant (rs235756) and the BMP4 gene variant (rs4901417) were associated with hypertension [14].

This study focused on the rs17563 of the BMP4 gene and the rs1005464 of the BMP2 gene. The rs1005464 SNP is located in the non-coding region of BMP2 and is classified as an intronic variant. Its molecular and metabolic pathways have not yet been fully elucidated, but it may influence splicing patterns or affect the rate, efficiency, and accuracy of gene transcription [26]. In this study, the rs1005464 was found to be associated with hypertension. Specifically, the GG genotype increases the risk of hypertension in women, non-elderly individuals, and alcohol drinkers. On the other hand, rs17563 of BMP4 is a missense variant that causes an amino acid substitution (V152A) [27], potentially affecting mRNA stability, protein expression, and receptor binding affinity [28], thereby altering BMP4 signaling [29]. Our study demonstrated that interactions between rs17563 (BMP4) and rs235756 (BMP2) influence hypertension risk. BMP2 and BMP4 proteins share similar signaling pathways, activating Smad proteins that regulate gene expression [30]. Their combined effects in VSMCs and endothelial cells may enhance cell proliferation and contractility, increase vascular resistance [31], and promote hypertension [32]. This synergistic interaction highlights the role of BMP-related genetic variations in hypertension pathogenesis.

Diseases are influenced by the combined effects of genetic and environmental factors. In this study, an interaction between the rs17563 polymorphism of the BMP4 gene and fluoride exposure was identified. Individuals with the AG + GG genotype and high fluoride exposure had over twice the risk of hypertension compared to those with the AA genotype and low fluoride exposure, suggesting a synergistic effect. The rs17563 polymorphism is associated with elevated serum BMP4 levels [33], which contribute to vascular calcification [34], endothelial dysfunction, and inflammation, all linked to hypertension [35,36]. Previous research shows that fluoride upregulates the BMP/Smad pathway [37], stimulating BMP gene expression and increasing serum BMP levels [38], which may enhance vascular calcification. Consistent with these findings, our study identified significant interactions between urinary fluoride concentration and the rs17563 polymorphism. We hypothesize that high fluoride exposure influences vascular calcification through the BMP pathway, providing new insights into the mechanisms underlying hypertension caused by fluoride exposure.

### Strengths and Limitation

It is noteworthy to mention that prior research has not documented any investigations on the correlation between gene polymorphism and fluoride exposure in relation to the susceptibility to hypertension. Consequently, the subject matter explored in this work possesses a distinctive and pioneering nature. The significance of the SNPs selected in this study on bone-related disorders has been demonstrated by thorough research in a wide range of individuals, so this postulates a mechanism: BMP2/4 gene mutations enhance hypertension risk with fluoride exposure. This work presents epidemiological evidence that vascular calcification, a link between BMP and hypertension, may play a regulatory function. Moreover, understanding the interplay between genetic polymorphisms and environmental exposures could lead to more personalized approaches in managing hypertension and other related conditions. However, this study has limitations. First, dietary and behavioral habits have a significant impact on hypertension, but the accuracy of the participants’ self-reported results is questionable and was therefore omitted from this study. Secondly, this study only included Han Chinese participants, and its findings may not be applicable to other ethnic groups. Finally, future research requires more varied and larger populations.

## 5. Conclusions

This study identified high fluoride exposure as a significant risk factor for hypertension. The rs1005464 (G > A) polymorphism of the BMP2 gene was associated with an increased risk of hypertension. Additionally, an interaction was discovered between the BMP2 rs1005464 polymorphism and the BMP4 rs17563 polymorphism in relation to hypertension. Furthermore, UF and the BMP4 rs17563 polymorphism have an interaction on hypertension.

## Figures and Tables

**Table 1:** Population characteristics of hypertension case group and control group.

	Case	Control	*p* ^c^	Test	OR(95%CI) ^d^
Age ^a^	63(54–67)	55(46–64)	**<0.001**	Z = −6.852	1.052(1.036–1.068)
BMI ^a^	26.421(23.980–28.611)	24.614(22.621–26.676)	**<0.001**	Z = −6.154	1.129(1.080–1.181)
Urinary fluoride(mg/L) ^a^	1.364(0.972–1.917)	1.147(0.825–1.726)	**<0.001**	Z = −3.665	1.207(1.006–1.499)
Gender ^b^					
Male	122(33.15%)	116(32.49%)			Ref.
Female	246(66.85%)	241(67.51%)	0.850	χ^2^ = 0.036	1.012(0.649–1.577)
Cigarette smoking ^b^					
No	290(78.80%)	295(82.63%)			Ref.
Yes	78(21.20%)	62(17.37%)	0.192	χ^2^ = 1.705	1.578(0.966–2.577)
Alcohol drinking ^b^					
No	304(82.60%)	288(80.67%)			Ref.
Yes	64(17.40%)	69(19.33%)	0.501	χ^2^ = 0.454	0.800(0.483–1.326)

Bold indicates statistical significance at 0.05 level. ^a^ Age, BMI, and urinary fluoride concentrations did not follow a normal distribution and are reported as medians with interquartile ranges (25%, 75%). ^b^ Number (percentage/proportion) for categorical variables. ^c^ the Mann–Whitney U test was applied to compare the difference of continuous variables, and chi-squared test was used to compare the difference of categorical variables. ^d^ Crude model.

**Table 2:** Association of urinary fluoride (mg/L) with hypertension.

UF Levels (mg/L, Median[Range])	N(EH/Non-EH)	Model1		Model2	
		OR(95%CI)	*p*	OR(95% CI)	*p*
Q1(0.70[<0.87])	227(92/135)	1.00		1.00	
Q2(1.08[0.88–1.25])	227(104/123)	1.352(0.892–2.049)	0.156	1.211(0.779–1.881)	0.395
Q3(1.48[1.26–1.80])	228(125/103)	2.063(1.358–3.134)	**0.001**	1.513(1.178–2.850)	**0.007**
Q4(2.39[≥1.81])	227(133/94)	2.017(1.328–3.063)	**0.001**	1.647(1.056–2.570)	**0.028**
Trend test		1.496(1.182–1.894)	**0.001**	1.343(1.045–1.726)	**0.021**
Increase per 1mg/L		1.307(1.098–1.556)	**0.003**	1.207(1.006–1.449)	**0.043**

Bold indicates statistical significance at 0.05 level. Model 1: crude model. Model 2: adjusted for age, gender, BMI, cigarette smoking, and alcohol drinking. Trend test: median UF for each quantile was included as a continuous variable.

**Table 3:** Five genetic models’ analysis of the association between the SNPs of BMP2/BMP4 and hypertension in all subjects.

SNP	Genetic Model	Genotype	Case N(%)	Control N(%)	OR(95%CI)	*p* ^a^	AIC	BIC
rs1005464	Codominant	GG	158(42.9)	127(35.6)	1	**0.044**	931.8	973.1
		AG	165(44.8)	186(52.1)	0.65(0.47–0.91)			
		AA	45(12.2)	44(12.3)	0.78(0.47−1.29)			
	Dominant	GG	158(42.9)	127(35.6)	1	**0.016**	930.3	967
		AG + AA	210(57.1)	230(64.4)	0.68(0.49–0.93)			
	Recessive	GG + AG	323(87.8)	313(87.7)	1	0.97	936.1	972.7
		AA	45(12.2)	44(12.3)	0.99(0.62−1.58)			
	Overdominant	GG + AA	203(55.2)	171(47.9)	1	**0.021**	930.7	967.4
		AG	165(44.8)	186(52.1)	0.69(0.51–0.95)			
	Log-additive	---	---	---	0.81(0.64−1.02)	0.076	932.9	969.6
rs17563	Codominant	AA	182(49.5)	184(51.5)	1	0.27	935.5	976.7
		AG	151(41)	148(41.5)	1.07(0.77−1.48)			
		GG	35(9.5)	25(7.0)	1.61(0.90−2.89)			
	Dominant	AA	182(49.5)	184(51.5)	1	0.38	935.3	972
		AG + GG	186(50.5)	173(48.5)	1.15(0.84−1.57)			
	Recessive	AA + AG	333(90.5)	332(93.0)	1	0.12	933.6	970.3
		GG	35(9.5)	25(7.0)	1.56(0.89−2.75)			
	Overdominant	AA + GG	217(59.0)	209(58.5)	1	0.99	936.1	972.7
		AG	151(41.0)	148(41.5)	1.00(0.73−1.37)			
	Log-additive	---	---	---	1.18(0.93−1.50)	0.18	934.2	970.9

Bold indicates statistical significance at 0.05 level. ^a^ Adjusted for age (continuous), gender, urinary fluoride (continuous), BMI (continuous), cigarette smoking, and alcohol drinking.

**Table 4:** Distribution of hypertension prevalence in the GG genotype group vs. the AG + AA genotype group at the rs1005464 of BMP2.

Variant	Control(N)	Case(N)	χ^2^	*p* ^a^
Total	357	368	4.115	**0.043**
Age < 60	223	155	4.152	**0.042**
Age ≥ 60	134	213	1.193	0.275
Male	116	122	1.576	0.209
Female	241	246	2.533	0.112
Urinary fluoride ≤ 1.6 mg/L	256	235	2.396	0.122
Urinary fluoride > 1.6 mg/L	101	133	1.466	0.226
BMI < 24	152	92	1.687	0.194
BMI ≥ 24	205	276	2.606	0.106
Cigarette Smoking	62	78	2.159	0.142
Non-Cigarette Smoking	295	290	2.316	0.128
Alcohol Drinking	69	64	9.179	**0.002**
Non-Alcohol Drinking	288	304	0.653	0.419

Bold indicates statistical significance at 0.05 level. ^a^ Adjusted for age (continuous), gender, urinary fluoride (continuous), BMI (continuous), cigarette smoking, and alcohol drinking.

**Table 5:** Association of BMP2 rs1005464 with hypertension risk stratified by potential risk factor.

	rs1005464	Control N (%)	Case N(%)	OR(95%CI)	*p* ^g^	*p* ^h^
Age ^a^						
Age < 60	GG	140(62.8)	75(48.4)	1(Ref)		
	AG + AA	83(37.2)	80(51.6)	0.582(0.376−0.900)	**0.031**	**0.015**
Age ≥ 60	GG	44(32.8)	83(39.0)	1(Ref)		
	AG + AA	90(67.2)	130(61.0)	0.808(0.500–1.305)	0.249	0.383
Gender ^b^						
Male	GG	43(37.1)	55(45.1)	1(Ref)		
	AG + AA	73(62.9)	67(54.9)	0.704(0.402–1.233)	0.210	0.220
Female	GG	84(34.9)	103(41.9)	1(Ref)		
	AG + AA	157(65.1)	143(58.1)	0.667(0.448−0.991)	0.112	**0.045**
Urinary fluoride ^c^						
UF ≤ 1.6 mg/L	GG	89(34.8)	98(41.7)	1(Ref)		
	AG + AA	167(65.2)	137(58.3)	0.690(0.467–1.021)	0.122	0.063
UF > 1.6 mg/L	GG	38(37.6)	60(45.1)	1(Ref)		
	AG + AA	63(62.8)	73(54.9)	0.625(0.349–1.120)	0.227	0.114
Body mass index (kgm^2^) ^d^						
BMI < 24	GG	55(36.2)	41(44.6)	1(Ref)		
	AG + AA	97(63.8)	51(55.4)	0.641(0.365–1.124)	0.195	0.121
BMI ≥ 24	GG	72(35.1)	117(42.4)	1(Ref)		
	AG + AA	133(64.9)	159(57.6)	0.684(0.460–1.017)	0.107	0.061
Cigarette Smoking ^e^						
Yes	GG	21(33.9)	36(46.2)	1(Ref)		
	AG + AA	41(66.1)	42(53.8)	0.497(0.234–1.055)	0.530	0.069
No	GG	106(35.9)	122(42.1)	1(Ref)		
	AG + AA	189(64.1)	168(57.9)	0.719(0.502–1.029)	0.150	0.071
Alcohol Drinking ^f^						
Yes	GG	17(24.6)	32(50.0)	1(Ref)		
	AG + AA	52(75.4)	32(50.0)	0.296(0.134−0.655)	**0.003**	**0.003**
No	GG	110(38.2)	126(41.4)	1(Ref)		
	AG + AA	178(61.8)	178(58.6)	0.815(0.570–1.165)	0.399	0.262

Bold indicates statistical significance at 0.05 level. ^a^ Adjusted for gender, urinary fluoride (continuous), BMI (continuous), cigarette smoking, and alcohol drinking. ^b^ Adjusted for age (continuous), urinary fluoride (continuous), BMI (continuous), cigarette smoking, and alcohol drinking. ^c^ Adjusted for age (continuous), gender, BMI (continuous), cigarette smoking, and alcohol drinking. ^d^ Adjusted for age (continuous), gender, urinary fluoride (continuous), cigarette smoking, and alcohol drinking. ^e^ Adjusted for age (continuous), gender, urinary fluoride (continuous), BMI (continuous), and alcohol drinking. ^f^ Adjusted for age (continuous), gender, urinary fluoride (continuous), BMI (continuous), and cigarette smoking. ^g^ Crude model. ^h^ Adjust model.

**Table 6:** The interaction effect of rs1005464 and rs17563 on EH.

rs1005464	rs17563	Control (N)	Case (N)	Model 1 ^a^		Model 2 ^b^		*p_-interaction 1_* ^a^	*p_-interaction 2_* ^b^
				OR(95%CI)	*p*	OR(95%CI)	*p*		
AG + AA	AA	110	109	Ref.		Ref.			
GG	AA	74	73	0.99(0.66–1.51)	0.983	0.97(0.62–1.51)	0.880		
AG + AA	AG + GG	120	101	0.85(0.58–1.24)	0.393	0.84(0.56–1.24)	0.376		
GG	AG + GG	53	85	1.62(1.05–2.50)	**0.029**	1.99(1.25–3.17)	**0.004**	**0.035**	**0.008**
	RERI			0.77(0.07–1.55)		1.13(0.33–2.16)			
	SI			0.15(0.01–1.99)		0.11(0.01–1.74)			

Bold indicates statistical significance at 0.05 level. ^a^ Crude model. ^b^ Adjusted for age, gender, UF, BMI, cigarette smoking, and alcohol drinking.

**Table 7:** The interaction effect of UF and BMP4 rs17563 SNP on hypertension.

UF ^a^ (mg/L)	rs17563 Genotype	Control (N)	Case (N)	Model 1 ^b^		Model 2 ^c^		*p_-interaction 1_* ^b^	*p_-interaction 2_* ^c^
				OR(95%CI)	*p*	OR(95%CI)	*p*		
≤1.6	AA	125	127	Ref.		Ref.			
≤1.6	AG + GG	130	108	1.23(0.86–1.76)	0.248	1.23(0.76–1.97)	0.398		
>1.6	AA	59	55	1.13(0.72–1.77)	0.602	1.06(0.65–1.7)	0.836		
>1.6	AG + GG	43	78	2.25(1.43–3.55)	**<0.001**	2.21(1.27–3.86)	**0.005**	**0.005**	**0.006**
RERI				−1.35(−2.51–0.2)		−1.38(−2.73–0.03)			
SI				0.09(0–3.26)		0.04(0–168.11)			

Bold indicates statistical significance at 0.05 level. ^a^ UF included as a continuous variable. ^b^ Crude model. ^c^ Adjusted for gender, age, BMI, cigarette smoking, and alcohol drinking.

**Table 8:** The interaction effect of UF and BMP2 rs1005464 SNP on hypertension.

UF ^a^ (mg/L)	rs1005464 Genotype	Control (N)	Case (N)	Model 1 ^b^		Model 2 ^c^		*p_-interaction 1_* ^b^	*p_-interaction 2_* ^c^
				OR(95%CI)	*p*	OR(95%CI)	*p*		
≤1.6	AG + AA	167	137	Ref.		Ref.			
≤1.6	GG	89	98	1.34(0.93–1.93)	0.114	1.43(0.97–2.11)	0.072		
>1.6	GG	38	60	1.92(1.21–3.06)	**0.006**	1.95(1.19–3.21)	**0.008**		
>1.6	AG + AA	63	73	1.41(0.94–2.12)	0.095	1.25(0.81–1.91)	0.309	0.963	0.794
RERI				0.17(−0.84–1.18)		0.29(0.79–1.35)			
SI				1.23(0.36–4.17)		1.41(0.36–5.49)			

Bold indicates statistical significance at 0.05 level. ^a^ UF included as a continuous variable. ^b^ Crude model. ^c^ Adjusted for gender, age, BMI, cigarette smoking, and alcohol drinking.

## Data Availability

The data that support the findings of this study are available from the corresponding author upon reasonable request.

## Supplementary Materials

The following supporting information can be downloaded at: https://www.mdpi.com/article/10.3390/toxics13020126/s1, Table S1: Comparisons of the characteristics between the incluided and excluded participants by blood pressure levels.
