# Supplementary material for: Interactions Between BMP2/BMP4 Gene Polymorphisms and Fluoride Exposure on Essential Hypertension: A Cross-Sectional Study in China
Source: Toxics. 2025 Feb 8;13(2):126. doi: 10.3390/toxics13020126 (PMC11860847; doi:10.3390/toxics13020126)
Supplement: Supplementary file 1 [file toxics-13-00126-s001.zip › toxics-3394757-supplementary.pdf]

Table S1. Comparisons of the characteristics between the incluided and excluded participants by blood pressure levels

Variables	Case group (n=502)			Control group (n=487)		
	Included (n=368)	Excluded (n=134)	*p* [c]	Included (n=357)	Excluded (n=130)	*p c*
Age[a] (years)	63(49,66)	63(55,68)	0.328	55(46,64)	53(45,63)f	0.473
Sex[b]			0.779			0.183
Male	122(33.2%)	53(39.6%)		116(32.5%)	44(33.8%)	
Female	246(66.8%)	81(60.4%)		241(67.5%)	86(66.2%)	
Body mass index (kg/m2)[a]	26.42(23.98,28.62)	26.01(23.91,28.62)	0.641	24.62(22.62,26.68)	24.49(22.66,27.35)	0.724
UF(mg/L)[a]	1.36(0.97,1.92)	1.49(0.90,2.27)	0.212	1.15(0.82,1.73)	1.28(0.87,1.87)	0.187
Cigarette smoking[b]			0.914			0.390
No	290(78.8%)	29(21.6%)		295(82.6%)	103(79.2%)	
Yes	78(21.2%)	105(78.4%)		62(17.4%)	27(20.8%)	
Alcohol drinking[b]			0.102			0.868
No	288(80.7%)	104(80.0%)		304(82.6%)	102(76.1%)	
Yes	69(19.3%)	26(20.0%)		64(17.4%)	32(23.9%)	

[a]Age, BMI, and urine fluoride concentrations did not follow a normal distribution and are reported as medians with interquartile ranges (25%, 75%).
[b]Number (percentage/proportion) for categorical variables.
[c]the Mann-Whitney U test was applied to compare the difference of continuous variables, and Chi-square test was used to compare the difference of categorical variables.
